# The Influence of Flaxseed Oil Cake Extract on Oxidative Stability of Microencapsulated Flaxseed Oil in Spray-Dried Powders

**DOI:** 10.3390/antiox10020211

**Published:** 2021-02-01

**Authors:** Emilia Drozłowska, Artur Bartkowiak, Paulina Trocer, Mateusz Kostek, Alicja Tarnowiecka-Kuca, Grzegorz Bienkiewicz, Łukasz Łopusiewicz

**Affiliations:** 1 Center of Bioimmobilisation and Innovative Packaging Materials, Faculty of Food Sciences and Fisheries, West Pomeranian University of Technology Szczecin, Janickiego 35, 71-270 Szczecin, Poland; Artur-Bartkowiak@zut.edu.pl (A.B.); p.trocer@gmail.com (P.T.); mkosa9406@gmail.com (M.K.); alicja.tarnowiecka-kuca@zut.edu.pl (A.T.-K.); lukasz.lopusiewicz@zut.edu.pl (Ł.Ł.); 2 Department of Commodity, Quality Assessment, Process Engineering and Human Nutrition, Faculty of Food Sciences and Fisheries, West Pomeranian University of Technology Szczecin, Papieża Pawła VI 3, 71-899 Szczecin, Poland; grzegorz.bienkiewicz@zut.edu.pl

**Keywords:** spray drying, flaxseed oil cake, emulsions, antioxidant activity, flaxseed oil, oxidative stability, α-linolenic acid

## Abstract

The objective of the study was to investigate the application of flaxseed oil cake extract (FOCE) for oxidative stabilization of flaxseed oil in spray-dried emulsions. Two variants of powders with 10% and 20% of flaxseed oil (FO), FOCE, and wall material (maltodextrin and starch Capsul^®^) were produced by spray-drying process at 180 °C. The oxidative stability of FO was monitored during four weeks of storage at 4 °C by peroxide value (PV) and thiobarbituric acid-reactive substances (TBARS) measurements. Additionally, the fatty acids content (especially changes in α-linolenic acid content), radical scavenging activity, total polyphenolics content, color changes and free amino acids content were evaluated. Obtained results indicated that FOCE could be an adequate antioxidant dedicated for spray-dried emulsions, especially with a high content of FO (20%). These results have important implications for the flaxseed oil encapsulation with natural antioxidant agents obtained from plant-based agro-industrial by product, meeting the goals of circular economy and the idea of zero waste.

## 1. Introduction

The cultivation and use of flaxseed (*Linum usitatissimum* L.) and its oil have been reported since antiquity [1]. However, in recent years, interest in flaxseed oil has rapidly grown due to the awareness of consumers about healthy eating and a balanced diet. Currently, flaxseed oil is used for industrial purposes, such as the production of paints, linoleum, varnishes, inks, and cosmetics. Additionally, it is used in food and pharmaceutical industries [1,2]. Flaxseed oil, unlike fish oil, does not contain EPA (eicosapentaenoic acid) or DHA (docosahexaenoic acid), however, it is still gaining the attention of consumers who are interested in a healthy diet [3]. Flaxseed oil (having cardioprotective and antiatherogenic activity) is appreciated by consumers due to the composition of fatty acids—large amounts (over 90%) of polyunsaturated fatty acids (PUFA), including α-linolenic acid (ALA)-ω-3 essential unsaturated fatty acid, consumption of which may have beneficial effects on health [4,5,6,7,8]. Health properties of ALA include the ability to reduce the blood pressure, to decrease the concentration of triacylglycerols in blood plasma, as well as anticoagulant and antitumor effects [9]. However, one of the major problems associated with PUFA-rich oils is their high susceptibility to oxidative deterioration (when exposed to oxygen, heat, or light), and additionally consequent production of undesirable flavor, which are not accepted in food industry [8,9]. Flaxseed oil also contains phospholipids, the major component of lecithins, which are a by-product isolated during the extraction of edible oils. The main phospholipids in flaxseed oil are lysophosphatidylcholine (LPC), phosphatidylethanolamine (PE), and phosphatidylinositol (PI). They are paid attention due to their health benefits [10].

Lipid oxidation starts a cascade of unfavorable biochemical changes occurring in products. Light radiation and atmospheric oxygen are factors that initiate lipid oxidation and generate free radicals supporting the process [9]. As a result of oxidation, primary harmful oxidation products are formed, such as peroxides, which then break down into toxic secondary oxidation products, such as carbonyl compounds, conjugated dienes, and furans [11]. Secondary oxidation products contribute to a loss in nutritional and organoleptic properties of food [9]. Lipid oxidation contributes to a reduction in the quality of food rich in fats, which is a serious problem in food storage and distribution [12]. Moreover, some products of oxidation are toxic and reduce the bioavailability of fatty acids. Oils with a high content of unsaturated fatty acids are more sensitive to oxidation in comparison to those with increased concentrations of saturated fatty acids [13]. Therefore, a very important aspect is preventing PUFA-rich oils from oxidation [1,8].

One of the wide range of possibilities for preventing the oils from oxidation is the use of a microencapsulation technique by spray drying [4]. Spray drying is a technique to obtain a final product in the form of a powder from the starting liquid raw material. The conversion of emulsions (containing PUFA) into powdered products, by the use of the spray-drying technique, represents a very promising solution to improve their oxidative stability. Moreover, encapsulation may increase the solubility of hydrophobic compounds in lipophilic systems [14].

The food industry generates a large amount of waste every year, which opens up a research field aimed at minimizing and efficiently managing this issue to support the concept of zero waste [15]. Oil cakes/oil meals are the by-products obtained after oil extraction from the seeds [15,16]. These residues are a source of bioactive compounds (protein, dietary fiber, antioxidants) with beneficial properties for health that can be used in foods. Another advantage is in the economic field: oil cakes are a cheap, safe material available throughout the year [15]. The use of oil seed press cakes could be a sustainable alternative to reduce waste disposal and also contributes to the development of new, low-cost products rich in nutrients. According to Fruehwirth et al. [17] during the cold pressing process the flaxseed polyphenols remain in the oil cake and only a small amount is transferred into the oil. The way to prevent flaxseed oil during spray drying process could be the addition of flaxseed oil cake extract (FOCE) as a liquid phase to the core of a microcapsule. The valorization of FOCE (a mixed liquid matrix of flaxseed protein and flaxseed gum, rich in antioxidants such as polyphenolics) is a relatively new issue, however, there are reports about its technological applications, including stabilization of FO oil-in-water emulsion systems and the production of spray-dried powders with emulsifying activity that indicate the potential of this valuable agro-industrial by-product with respect to the circular economy and sustainable development [18,19,20]. The idea of the utilization of flaxseed products and by-products has been already reported by Hano et al., who used secoisolariciresinol extracted from FOCE to stabilize flaxseed oil [8]. Moreover, the preparation of emulsions with natural emulsifiers and antioxidants is effective, and allows for avoiding the possible toxicity of synthetic compounds [1,8,21].

To the best of our knowledge there is a lack of reporting about the oxidative stability of flaxseed oil in spray-dried emulsions with flaxseed by-products using a natural antioxidant and stabilizer. Thus, the aim of the presented study is the investigation of FOCE as an antioxidant agent for the spray drying of emulsions containing FO. The powders with various content of flaxseed oil were prepared and their physico-chemical properties (including FO oxidative stability) during cold storage for four weeks were evaluated.

## 2. Materials and Methods

### 2.1. Materials

Flaxseed oil cake (FOC) obtained via cold-press technique was kindly donated by ACS Sp. Z o.o. (Bydgoszcz, Poland). Starch Capsul^®^ (National Starch and Chemical, Bridgewater, MA USA), maltodextrin (PEPEES S.A., Łomża, Poland), and flaxseed oil (Olandia, Prusim, Poland) were also used. According to the manufacturer’s information the proximate composition of FOC was as follows: 80.5% solids content, 42% protein content, 28% carbohydrates content, 6.3% fiber content, 6.1% fat content, 4.5% ash content. Sodium hydroxide, phenolphthalein, 2,2-diphenyl-1-picrylhydrazyl (DPPH), 2,2′-azino-bis(3-ethylbenzothiazoline-6-sulfonic acid) (ABTS), methanol, potassium persulfate, Folin-Ciocalteu’s reagent, sodium carbonate, gallic acid, ninhydrin, glacial acetic acid, cadmium chloride, glycine, trichloroacetic acid (TCA), 2-thiobarbituric acid (TBA), 1,1,3,3- tetramethoxypropane, sodium tiosulphate, and potassium iodide were purchased from Sigma Aldrich (Darmstadt, Germany). Hydrochloric acid, chloroform, n-hexane and ethanol were supplied from Chempur (Piekary Śląskie, Poland). The internal standard for gas chromatography nonadecanoic acid C:19 was purchased from Merck Chemical (Saint Louis, MO USA). All reagents were of analytical grade.

### 2.2. Formulation of Emulsion and Spray Drying Process

The procedure to obtain flaxseed oil cake extract (FOCE) (used as a liquid phase) was carried out following the procedure described in previous study [19]. Starch Capsul^®^ and maltodextrin were used as a wall material in ratio 1:1 (8.5 g of each per 100 mL of FOCE). The wall components were added to FOCE at 25 °C and the mixture was stirred (250 rpm) until the solids were completely dissolved (approx. 15 min). Flaxseed oil (FO) was then added to the solution at a concentration of 10% and 20% with respect to total solids content of mixture. The model emulsions were prepared in two steps. In the first step the mixtures were mixed with FO for 5 min (500 rpm) with a magnetic stirrer (IKA, Staufen, Germany). The next step included homogenization for 5 min with a homogenizer (Magic LAB UTC, IKA, Staufen, Germany) at 1500 rpm. Powdered FO emulsions were obtained by spray-drying using a lab-scale spray dryer (Büchi B-290, Büchi Labortechnik AGT, Flawill, Switzerland). The drying air inlet temperature of 180 °C was chosen based on the results described elsewhere [20]. The drying air outlet temperature was maintained at 55 ± 5 °C. Dried powders were collected in a glass collection vessel and stored tightly closed in darkness at 4 °C and stored for four weeks.

### 2.3. Powder Characterization

#### 2.3.1. Water Activity, Hygroscopicity and Particle Size of Powders

The analyses of water activity, hygroscopicity, and particle size of powders (D_4,3_) were carried out directly after the spray drying process. Water activity (a_w_) was measured at 25 °C using MS1 Set-aw (Novasina, Lachen, Switzerland) equipment. The emulsions’ powdered samples (approx. 1 g) were placed in the device and left for 30 min to stabilize, then measured for a_w_. Hygroscopicity was determined based of the methodology of Cai and Corke [22] with a slight modification. One gram of the powders was placed in a Binder climate chamber (Binder, Tuttingen, Germany) at 25 °C (70.00% RH). The samples were weighted after one week. Hygroscopicity was determined as the percent of moisture adsorbed per 100 g of dry solids. The particles size distribution of the spray-dried powders was determined using a Mastersizer 2000 with a Scirocco 2000 dry sampling system (Malvern Instrument Ltd., Worcestershire, UK). Procedure parameters were as follows: refractive index: 1.52, vibration feed rate: 50%, measurement time: 10 s, dispersive air pressure: 4 bar. The size measurements were described as the volume-weighted mean diameter D_4,3_ = ∑n_i_d_4i_/∑n_i_d_i3_. Each sample was measured in triplicate.

#### 2.3.2. Encapsulation Efficiency and Entrapment Oil

The encapsulation efficiency (EE) was expressed as the amount of oil entrapped in powders according to Calvo et al. [23]. Briefly, 5 g of each powder was precisely weighed into a Falcon tube and 50 mL of n-hexane was added. Then the mixture was shaken for 15 s at 25 °C to extract superficial oil. Next, the solvent mixture was filtered throughout a filter paper and after that unencapsulated surface oil was collected after vacuum n-hexane evaporation. The total oil content of powders was determined by Soxhlet protocol with a B-811 extraction system (Büchi Labortechnik AGT, Flawill, Switzerland) and calculated based on the following formula [23]:

(1) $$\mathrm{EE}\;\left(\%\right)=\frac{\mathrm{TO}-\mathrm{SO}}{\mathrm{TO}}\times 100$$

where TO is the total oil content, and SO is the unencapsulated oil content. Each sample was measured in triplicate.

### 2.4. pH Measurements and Titratable Acidity

pH and titratable acidity (TA) were carried out directly after spray drying process and after 1, 2, 3, and 4 weeks of storage at 4 °C. For the pH measurements the powders were dissolved in distilled water in ratio 1:10 *w*/*w*. Samples were measured directly at 25 °C using a pH meter (CP-411, Elmetron, Zabrze, Poland). The titratable acidity (TA) determination in the dissolved powder was performed according to Chegini et al. [24] with a slight modification. The one milliliter of sample was mixed with 10 mL of distilled water and titrated with 0.01 M NaOH solution, using phenolphthalein (0.1%, *w*/*v* in 96% ethanol) as an indicator. TA was calculated according the formula:

(2) $$\mathrm{TA}\;\left(\%\right)=\frac{v\times N\times 90\times 100}{V\times 1000}$$

where v is volume of 0.01 M NaOH, N is the normality of 0.01 M NaOH, and V is the volume of the sample solution (mL). Each sample was measured in triplicate.

### 2.5. Color Measurements

All powders were measured for color during the storage time by a Konica Minolta CR-5 colorimeter (Konica Minolta, Osaka, Japan). The values measured were L* (white 100/black 0), a* values (red positive/green negative), and b* values (yellow positive/blue negative). Each sample was measured in triplicate. The Whiteness Index (WI), Yellowness Index (YI), and ΔE were calculated according the following formulas [18]:

(3) $$\mathrm{WI}=100-{\left[\left(100-L^{*}\right)+a^{2}+b^{2}\right]}^{0.5}$$

(4) $$\mathrm{YI}=142.86\times b\times L^{-1}$$

(5) $$\Delta E={\left[{\left(L_{\mathrm{standard}}-L_{\mathrm{sample}}\right)}^{2}+{\left(a_{\mathrm{standard}}-a_{\mathrm{sample}}\right)}^{2}+{\left(b_{\mathrm{standard}}-b_{\mathrm{sample}}\right)}^{2}\right]}^{0.5}$$

### 2.6. FTIR Analyses

The FTIR spectra of the emulsion powders were obtained at 25 °C by attenuated total reflection with an FTIR spectrometer (Perkin Elmer Spectrophotometer 100, Waltham, MA, USA). The powders after each week of storage (100 mg) were scanned at a range between 650 cm^−1^ and 4000 cm^−1^ (100 scans and 4 cm^−1^ resolution). The obtained spectra were normalized, baseline corrected, and analyzed using SPECTRUM software (v10, Perkin Elmer Spectrophotometer, Waltham, MA, USA).

### 2.7. Antioxidant Activity, Total Free Amino Acids, and Total Polyphenolic Content of Powders

The antioxidant activity, total polyphenolic content (TPC) and total free amino acids (TFAA) of the powders were analyzed directly after spray drying process and after 1, 2, 3, and 4 weeks of storage at 4 °C. The DPPH and ABTS radical scavenging activity were determined according to the procedures described elsewhere [25,26]. Firstly, the powders were diluted in distilled water (1:100 *w*/*v*), vortexed (5 min), then centrifuged at 14,000 rpm/min for 10 min at 20 °C (Centrifuge 5418 Eppendorf, Warsaw, Poland), and filtered through a 0.22 μm nylon membrane filter (Sigma-Aldrich, Darmstadt, Germany). The obtained clear fluids were used for further analyses. In brief, the DPPH radical scavenging activity was determined by mixing 1 mL of supernatant with 1 mL of 0.01 mM DPPH methanolic solution. The absorbance was measured at 517 nm. Three milliliters of ABTS·^+^ solution were mixed with 50 µL of the supernatant and the absorbance was measured at 734 nm.

Total free amino acids level (TFAA) was analyzed according to Barac et al. [27] with a slight modification. A quantity of 1 mL of supernatants were mixed with 2 mL of a Cd-ninhydrin reagent (0.8 g ninhydrin was dissolved in a mixture of 80 mL of 96% ethanol and 10 mL glacial acetic acid, followed by the addition of 1 g CdCl_2_ dissolved in 1 mL of distilled water). The samples were vortexed and heated at 84 °C for 5 min and cooled in ice-water and the absorbance was determined at 507 nm. The results were expressed as mg Gly per gram of sample by reference to a standard curve taking into account dilution factor. The standard curve was first prepared using glycine at various concentrations (0.050–0.500 mg Gly/mL water).

The supernatants for TPC measurements were obtained by dissolving powders in ratio 1:10 (*w*/*v*) and following the same centrifugation and filtration procedure. TPC was determined by Folin-Ciocalteu method as described by Tong et al. [28]. One hundred µL of supernatants were added to with 6 mL of distilled water and 0.5 mL of Folin-Ciocalteu’s reagent. After 3 min incubation, 1.5 mL of saturated Na_2_CO_3_ solution was added. In the next step the mixtures were incubated for 30 min in darkness at 40 °C. The absorbance of the mixtures was measured at 765 nm (UV–VIS Thermo Scientific Evolution 220 spectrophotometer). The TPC was expressed as mg of gallic acid equivalents (GAE) per mL of sample (mg GAE/mL).

### 2.8. Oxidative Stability of Flaxseed Oil during Storage

Chloroform-methanol (2:1 *v*/*v*) extraction was performed on the homogenized products based on method of Blight and Dyer [29]. Next, in the chloroform phase the peroxide value (PV) was determined, as described elsewhere [30]. Analyses of the initial flaxseed oil were carried out for comparison.

#### 2.8.1. Secondary Oxidation Compounds

Every week the secondary oxidation compounds in extracted oil were measured. Thiobarbituric acid-reactive substances (TBARS) were determined according to Fioramonti et al. [31]. One mL of samples was combined with 2 mL of TBA-TCA (20 mM TBA in 15% (*w*/*v*) TCA) reagent and placed in a boiling water bath for 15 min. After cooling (25 °C) the samples were centrifuged (6000 rpm, 10 min). The absorbance was measured at 532 nm. The concentration of TBARS was determined by reference to a standard curve which was first prepared using 1,1,3,3-tetramethoxypropane.

#### 2.8.2. Detection of Fatty Acids and α-Linolenic Acid

Fatty acids (FA) and α-linolenic acid contents from extracted flaxseed oil were analyzed using gas chromatography, coupled with a mass spectrometer Agilent Technologies 7890A (Agilent Technologies, Santa Clara, CA, USA) and equipped with a split/spitless type injector. The separation was conducted with column SPTM 2560, 100 m 0.25 mm ID, 0.20 μm film thickness, catalogue no. 24056; carrier gas-helium at a constant flow rate of 1.2 mL/min; split 1:50; injector temperature: 220 °C; detector temperature: 220 °C; programmed furnace temperature: 140 °C (5 min) increased to 240 °C at a rate of 4 °C/min; analysis time: 45 min. The qualitative interpretation of chromatograms was based on the comparison of retention times and mass spectra of the particular fatty acids. C 19:0 was used as an internal standard.

### 2.9. Statistical Analyses

The measured parameters were subjected to one-way analysis of variance (ANOVA) using the statistical package with the software Statistica 13.0 (StatSoft, Kraków, Poland). Results are expressed as the mean ± standard deviation. Significant differences between means were determined by Fisher’s LSD (least significant difference) NIR multiple comparison tests at *p* < 0.05. All experiments were replicated three times. The trend line was generated with the software GraphPad Prism 8.0.1 (GraphPad Software, San Diego, CA, USA).

## 3. Results and Discussion

### 3.1. Water Activity (a_w_), Hygroscopicity, Particles Size of Powders and Encapsulation Efficiency of Flaxseed Oil

Average values of water activity (a_w_), hygroscopicity, particles size of powders, and encapsulation efficiency of flaxseed oil are summarized in Table 1. Significantly higher a_w_ was noticed for powder A containing 10% of FO (*p* < 0.05). Similar a_w_ values of spray-dried FOCE powders were observed in a previous study. When FOCE was dried at 180 °C and 200 °C, a_w_ of 0.48 and 0.36 was noticed, respectively [20]. However, the obtained results are higher than reported by Vélez-Erazo et al. for spray-dried vegetable oil emulsions stabilized by WPC (whey protein concentrate) and WPC-pectin systems [13]. Water plays a particularly significant role in lipid oxidation. According to Aberkane et al. [32] the low a_w_ protects the sensitive compounds, such as PUFA oils, against undesirable reactions. In the most food products this value is between 0.2 and 0.4, and lipid oxidation could increase rapidly when the a_w_ is nonstable and increases [33]. Similarly, a significantly higher hygroscopicity was observed for powder A (15.48 ± 0.88%) (*p* < 0.05). A similar values in the range of 15.87–18.90% was reported when gum Arabic was used as the wall material for rosemary oil encapsulation [34]. Lee et al. [35] suggested that influence of maltodextrin on hygroscopicity is correlated with many factors such as composition of wall material and the core of microcapsule. However, based on the criteria of Samsu et al. [36] the powders could be categorized as non-hygroscopic (less than 20%).

In the case of the average particle size of powders it could be observed that this parameter significantly increased with increased oil content, and the powder with 20% of FO presented higher average particles size (31.55 ± 0.67 µm; *p* < 0.05). However, this result is almost two-fold higher than reported by Vélez-Erazo et al. [13]. According to Chang et al. [37] the larger size of powders may exhibit a protective effect against oxidation. Chegini et al. [24] observed that the increase of particle size could be connected with the inlet air temperature. When the inlet temperature is optimal the top surface dries quickly. Thus, it forms a hard layer which does not allow internal moisture to leave the particle. The strong wall of microcapsules is very important during long-term storage. However, the higher particle size could be also connected with the higher oil volume.

A significantly higher EE (informing about the yield of encapsulation process) of sample B (39.00 ± 0.02%) in comparison with sample A (22.00 ± 3.24%) was observed (*p* < 0.05). These results are comparable with the results reported for squalene encapsulated in chitosan [38]. However, the obtained lower EE of powder A might suggest a high volume of the surface oil in the powder, which could be sensitive for oxidation through contact with oxygen [4,39]. Barbosa-Cánovas et al. [40] reported the correlation between emulsions stability, encapsulation efficiency, and surface oil. The authors suggested that when the emulsion exhibited high stability this parameter increased. Similarly, Tonon et al. [41] reported that the EE increased linearly with emulsions viscosity. Badke et al. [42] suggested that the high hydrophobic core must be enriched with surfactant. In fact, as reported in previous study, FOCE exhibited a tendency to more effectively stabilize emulsions with high content of FO. Moreover, the emulsifying and stabilizing activity of FOCE is linked with synergistic oil binding and water holding abilities of its two main components, flaxseed gum and flaxseed protein [19,20].

### 3.2. The Changes of pH and Titrable Acidity of Powders during Storage

**Figure 1:**
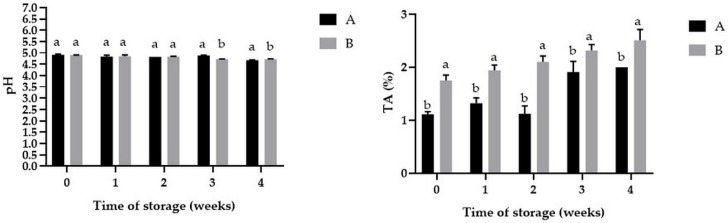
Changes of pH and TA during the storage in powders with 10% (A) and 20% (B) content of FO. Means with different lowercase letters are significantly different at *p* < 0.05.

The results of pH and titratable acidity measurements are presented in Figure 1. It was noticed that, the pH value was stable during the storage and first statistical important differences between samples (*p <* 0.05) were observed after three weeks of storage. Moreover, the increase of TA was observed in both samples, powder with 20% of FO showed a higher TA than powder with 10% of FO (*p* < 0.05) which could be attributed to higher level of FO. It was noticed that, during storage, the TA of powder A increased from 1.32 ± 0.10% to 2.00 ± 0.01%, whereas the TA of powder B increased from 1.94 ± 0.10% to 2.51 ± 0.20% (*p* < 0.05). This might suggest the interaction between various constituents of powders and the resulting chemical changes. The increase in TA may be due to the reaction during oil oxidation (hydrolysis and fatty acids forming) and to the degradation of sugars during the Maillard reaction [43,44]. The acidity of the environment is one of the important factors that can cause oxidation of oils. According to Kim et al. [45] lower pH may decrease the rate of lipid oxidation due to the easy transfer of protons to alkyl, alkoxy, and/or peroxyl lipid radicals formed during autoxidation.

### 3.3. Color Changes

The results of color measurements are listed in Table 2. Generally, powder B exhibited a higher yellowness index than powder A, which may be attributed to the higher content of oil. On the other hand, the lower YI of powder A could be an effect of maltodextrin which is usually bright white in its natural form [46]. The maltodextrin was also influenced by the lightness of samples, what was observed as higher L* of powder A during storage. A significant tendency to decrease of the b* parameter was noticed in both samples during storage (*p* < 0.05), indicating an increase of their yellowness. Indeed, significantly higher YI was noticed in both samples after four weeks when compared with fresh powders (A: 13.46 ± 0.08 and B: 16.72 ± 0.01). According to Klinkerson et al. [33] the products of lipid oxidation could polymerize and produce brown-colored oxypolymers in the presence of other compounds, such as amines, amino acids, and antioxidants. Thus, these reactions can lead to inactivation of free radicals. It is a pathway to produce significant color formation and oxidation process in powders [33]. Another explanation of higher yellowness of the samples be formation of Maillard reaction products after spray drying or polyphenolic-protein complexes [20]. Up to the third week the ∆E of both samples was lower than 1 and higher values were observed for sample A (*p* < 0.05). In the fourth week the highest ∆E (1.06 ± 0.05) was observed for powder A. When ∆E is higher than 1 the difference is considered perceptible to the human eye [20]. This parameter is a visualization of all color coordinate changes in the samples.

### 3.4. The Changes in Powder Chemical Composition

**Figure 2:**
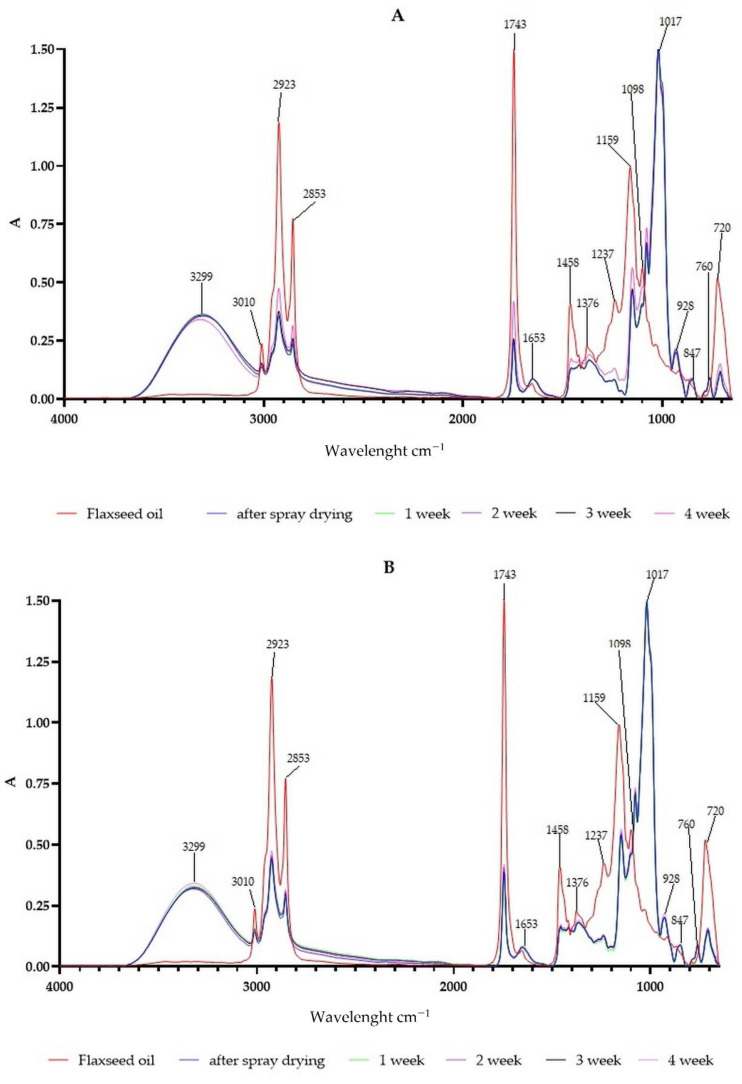
FTIR spectra of fresh flaxseed oil, powders with 10% (**A**) and 20% (**B**) and flaxseed oil, directly after spray drying, and after 1, 2, 3, and 4 weeks of storage.

FTIR spectroscopy was used to investigate changes in the structure and chemical composition of the powders caused by spray drying and storage. Absorbance spectra are presented in Figure 2. Slight differences could be observed between powders during storage. The peak at 1743 cm^−1^ comes from flaxseed oil. In the case of both samples the increase of this peak after three weeks was observed. Especially in sample A this signal was higher after four weeks, indicating oil release from the capsule. When the peak is lower for microcapsules than for oil, it is an effect of the integration of flaxseed oil in microcapsules and the presence of alkane group with C=C stretch [47]. According to Grehk et al. [48] the decrease of peaks at 3010 cm^−1^ (C-H stretching of aliphatic -CH=CH- related to unconjugated cis-double bonds) is correlated with cis-trans isomerization. It is a result of the oxidation and polymerization of flaxseed oil. This peak was lower in both cases in comparison to powders and, additionally, there was an observed decrease of it after three weeks of storage. It was also observed that the characteristic CH_3_ and CH_2_ stretching peaks at 2923 cm^−1^ and 2853 cm^−1^, respectively, which were previously described in the cases of flaxseed oil flaxseed gum, flaxseed protein, and FOCE [19]. The peak 1653 cm^−1^ in oil and powders is related to the carboxyl groups and double bond of oleic acid and ALA, respectively [49]. In case of powders this peak was higher than for oil and only negligible differences were observed during storage. This could be an effect of residual oil content in FOCE, previously described [19]. Peaks recorded at 1017 cm^−1^ (angular deformation of =CH and =CH_2_ bonds), 847 cm^−1^ (deformation of C-H and CH_2_), and peak 707 cm^−1^, (which is covered by peak 720 cm^−1^ from flaxseed oil) were previously described for the FOCE matrix [20]. Peak between 1098 and 1017 cm^−1^ is due to the presence of alkyl halide C=C stretch and peak at 1376 cm^−1^ comes from carboxylic acid R-CO∙O structure with C=O stretch. It was observed that signal from carboxylic acid group is absorbed at a similar wavelength. The peak at 1653 cm^−1^ from powders and flaxseed oil (C=C stretch) was also absorbed at the same wavelength.

### 3.5. The Changes of Antioxidant Activity, Total Free Amino Acids, and Total Polyphenolic Content

As can be seen in Table 3, the decrease of DPPH and ABTS scavenging activity, as well as total polyphenolic content was noticed for both powders during storage (*p* < 0.05). A similar effect was observed for spray-dried plant extracts [50] as well as spray-dried FOCE powders [20]. However, the antioxidant activity, as well as TPC, remained at relatively high levels and are comparable with the results reported for FOCE spray-dried powders [20]. Malcolmson et al. [51] indicated a lack of oxidative degradation in ground flaxseed stored at room temperature for several months. This phenomenon was explained by the presence of a potent antioxidant system which effectively protected flaxseed oil in situ from oxidation. Flaxseed is a rich source of phenolic compounds, such as lignans (secoisolariciresinol diglycoside (SDG)) and phenolic acids [52]. SDG is a diphenolic that is conjugated with mucilage and is water soluble. SDG and its aglycone (SECO) are the major lignans of flaxseed, showing antioxidant and efficient chemopreventive properties and having good thermal stability [53,54]. Moreover, free SDG is also present in flaxseed, and the way of processing and conditions (water extraction, spray-drying) can contribute to its level [55,56,57]. FOCE includes a mix of lignans, polysaccharides, and proteins, which could be affected by high temperature (protein denaturation or formation the new complexes between different compounds of FOCE) and bioavailability of particular components. The antioxidant activity is highly correlated with the hydrophobic properties of polyphenols [58]. The main lignans of flaxseed SECO and SDG exhibit lower antioxidant efficiency with high hydrophilic properties [59]. The high content of oil of presented samples (10% and 20%) and low water activity of powders could also have an influence on the initial scavenging activity of samples. It was noticed that powder B exhibited higher ABTS scavenging activity than powder A during storage (*p* < 0.05). The observed values of ABTS, generally higher than for DPPH, could be connected to different mechanisms of activity, which is based on the transfer of electrons and hydrogen atoms. In this case ABTS more precisely evaluates the antioxidant activity of both hydrophilic and lipophilic compounds [60]. It should be highlighted that, in the case of colloidal systems, ABTS does not depend solely on the amount of encapsulated antioxidant compounds, but also on the accessibility to the hydrophobic antioxidants compounds in redispersed powders [61].

The highest TPC was observed for initial samples (A: 19.08 ± 0.01 mg GAE/mL and B: 20.05 ± 0.01 mg GAE/mL). After four weeks sample A exhibited lower TPC (15.06 ± 0.00 mg GAE/mL) than sample B (16.98 ± 0.02 mg GAE/mL) (*p* < 0.05). The phenolic compounds included in FOCE are probably bounded in the microcapsules shell, which was formed during the spray drying [24]. This layer might protect both oil and polyphenols included in the core and entail higher antioxidation stability. Usually, the hot air drying may result in the loss of bioactive compounds. On the other hand, the spray drying process can activate the polyphenols’ ability to protect from oil oxidation. According to Mrkìc et al. [62] the oxidation process of polyphenols which takes place during drying could exhibit a positive influence because polyphenols partially oxidated might possess higher radical scavenging activity than non-oxidized polyphenols.

Free amino acids were also determined because of their role in product quality during processing, storage and likely in FO stabilization. A significantly higher initial content of TFAA after spray drying was observed in powder B (9.03 ± 0.13 mg Gly/mL) (*p* < 0.05). An increasing tendency of TFAA content was observed in both samples during storage (*p* < 0.05) and the highest TFAA was noticed for sample B after four weeks (15.50 ± 0.12 mg Gly/mL). The effect of amino acids on the oxidation of edible oils is still not well described. Oxidation of proteins included in product might lead to degradation of the polypeptide chain and to formation of cross-linked protein aggregates [63]. Additionally, the functional groups of proteins can react with components from the lipid oxidation process, such as hydroperoxides or aldehydes. Many authors reported several mechanisms of antioxidant activities of free amino acids which migrated to oil. These mechanisms include different processes, such as synergism with tocopherols, increase of radical scavenging ability, chelation of metals, and antioxidant activity of substances produced by reactions between amino acids and oxidized lipids, but these mechanisms are uncertain at this moment [63,64]. Additionally, the aim of the used protein mixes with polysaccharides in the case of oil encapsulation is the formation of Maillard conjugates which exhibit antioxidant mechanisms. Maillard conjugates, which are produced in the initial step of spray drying, could be stable for several days and influence the oxidative stability of microencapsulated oil [9,65]. Moreover, it is reported that hydrophobic amino acids have better reaction with free radicals due to greater solubility in lipids. Flaxseed is a source of amino acids with antioxidative properties [66]. Tryptophan, histidine, tyrosine, lysine, and methionine, which are included in flaxseed protein, are known as antioxidants [67]. These amino acids can result in scavenging of free radicals and reduction of oxidation rate through electron or proton-donating ability [68,69]. For instance, Martin-Rubio et al. [63] reported the ability of methionine to reduce lipid hydroperoxides to hydroxides. Spray drying is known to induce thermal denaturation of proteins and their partial degradation, resulting in change protein–protein hydrophobic, electrostatic, hydrogen-bonding, and disulfide-sulfhydryl interactions, which may affect the antioxidant activity of protein-rich extracts [20,70,71]. Thus, it is reasonable to conclude that denaturation and partial degradation of FOCE proteins presumably played a pivotal role in the stabilization of FO in spray-dried powders.

### 3.6. Oxidative Stability of FO and Changes of Fatty Acid Content

**Figure 3:**
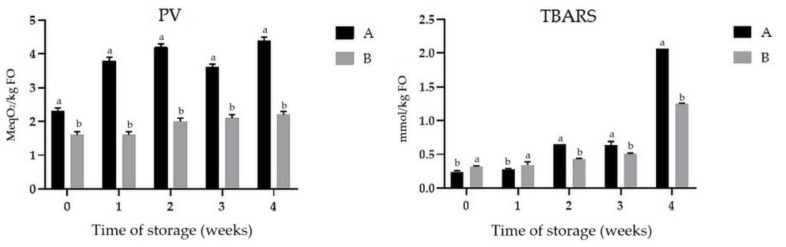
Peroxide values (PV) and thiobarbituric acid-reactive substances (TBARS) in powders with 10% (**A**) and 20% (**B**) content of FO. Means with different lowercase letters are significantly different at *p* < 0.05.

Oxidative stability is an important factor concerning oil microencapsulation [13].The progress of lipid oxidation was monitored by measuring the formation of primary (lipid hydroperoxides) and secondary reaction products. Peroxide value (PV) and TBARS in powders are presented in Figure 3. The PV of pure FO was 0.80 ± 0.01 meq O_2_/kg, whereas the TBARS level was 0.11 ± 0.02 mmol/kg. It is suggested that, at time zero, the formation of oxidation products could be related not only to spray drying, but additionally to oxygen inclusion during emulsion preparation [72]. Evaluation of PV is very important in the case of the spray drying process because a high inlet temperature might provide more energy for the lipid oxidation process to occur [25]. After four weeks the lipid oxidation PV values were 4.40 ± 0.05 meq O_2_/kg and 2.20 ± 0.00 meq O_2_/kg for samples A and B, respectively (*p* < 0.05). This indicates that FO in powder B (with higher oil content) showed higher oxidative stability. It is noteworthy that FOCE exhibited a tendency to more effectively stabilize emulsions when the oil volume was higher, as reported in previous study [19]. Fioramonti et al. [31] reported 6.8 meq O_2_/kg PV value for 18% flaxseed oil microencapsulated with whey protein concentrate (WPC), maltodextrin, and sodium alginate. It indicated that FOCE could prevent flaxseed oil from oxidation more effectively than WPC. According to Codex Alimentarius the level of lipid oxidation in vegetable oils, measured as PV, should not exceed 15 meq O_2_/kg oil for cold pressed and virgin oils [73]. Karaca et al. [39] stored flaxseed oil for 25 days and finally observed a PV of 12.91 ± 0.40 meq O_2_/kg. These results were close to the limit of PV described in Codex Alimentarius, and higher than observed in the present study. TBARS results were in line with PV results. This test measured the concentration of relatively polar secondary lipid oxidation products, especially aldehydes, including malondialdehyde (MDA) [74]. The higher formation of TBARS during storage was noticed for sample A with a lower FO content (*p* < 0.05). According to Fioramonti et al. [31] TBARS should be less than 1 mmol/kg oil. In fact, the level of secondary oil oxidation products in both powders did not exceed this value up to three weeks. However, some increase was observed on week 4. Thus, it is reasonable to conclude that FO in the powders was generally oxidatively stable, taking into account PV and TBARS, and it can be attributed to antioxidant properties of the wall material and core components of FOCE.

**Figure 4:**
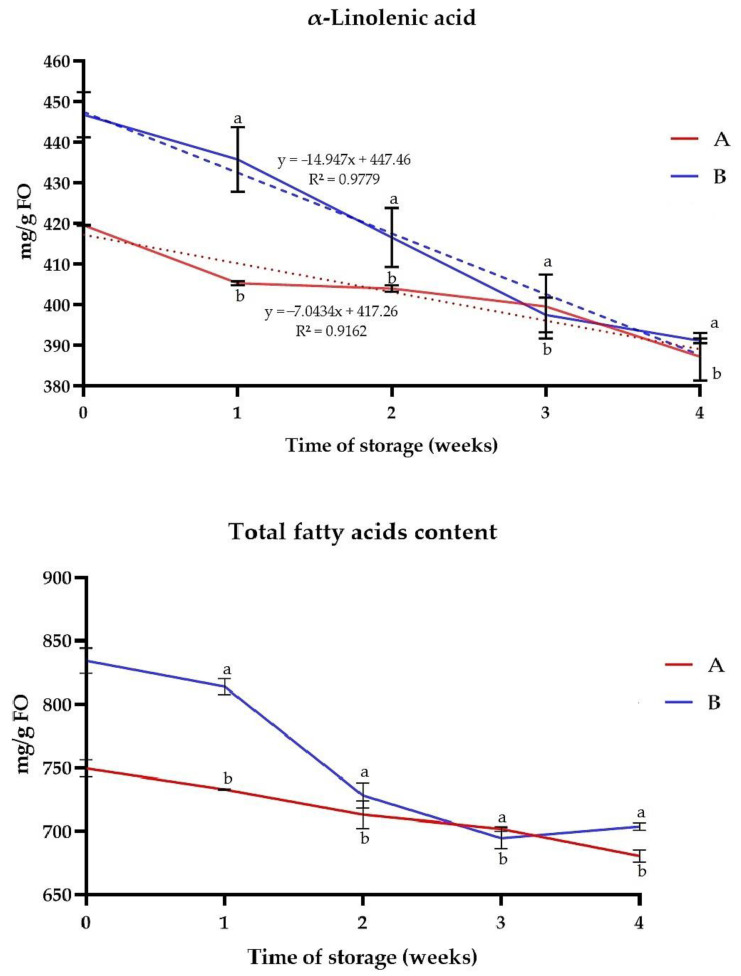
Changes of ALA and fatty acids content in powders containing 10% (**A**) and 20% (**B**) of FO. Means with different lowercase letters are significantly different at *p* < 0.05.

The changes of ALA and FFA in flaxseed oil extracted from the powders are presented in Figure 4. The initial level of ALA after the spray drying process was higher in powder B (446.78 ± 5.95 mg/g) than in powder A (419.61 ± 0.13 mg/g) (*p* < 0.05). ALA is the most important fatty acid of FO, which should be prevented against oxidation, and due to its low oxidative stability could be used as a marker of oxidative changes in powders [75]. The highest dynamic of ALA loss was observed until week 2 of storage. During the storage the loss of ALA was two times faster in powder B than in powder A, as expressed in Figure 4 with the trends lines. The “a” coefficient of the trend line was estimated respectively on 14.95 for the powder B (with 20% of FO) and 7.04 for sample A (with 10% of FO). This was indicated by the faster changes of ALA content in sample B. A similar tendency was observed regarding FFA level in powders. It could be concluded that the main factor during the oxidation process in powders was the amount of the ALA, because it was the substrate for this process. According to Goyal et al. [5] ALA is 20 times more susceptible to oxidation than oleic acid. However, Mao et al. [76] described that oleic acid could also play a role of limitation factor in oxidation process, such as in the presented study. The authors claimed that oxidation is closely related to the content of unsaturated fatty acids [76]. After the two weeks of storage the amount of primary oxidation products is stabilized at certain levels. After four weeks the amount of the main substrate (ALA) was equal and was noticed at approximately 400 mg ALA/g oil. The decrease of ALA and a decreasing total amount of fatty acids during storage was correlated with an increase in secondary oxidation products determined by the TBARS method (Figure 4). These products are formed in the process of peroxidation of polyunsaturated acyl groups or fatty acids [63,77]. Taking into account whole aforementioned results, the observed stability of FO can be also explained by polar paradox theory, which claimed that nonpolar antioxidants are more effective in relatively more polar media, such as oil-in-water and polar antioxidants are more effective in less polar media, such as bulk oils [78,79,80]. This could explain the observed phenomenon that sample with 20% of flaxseed oil exhibited better stability than the sample with 10%. SDG included in FOCE represents hydrophilic character and is able to be a better antioxidant in bulk oil [1,8]. According to Shahidi and Zhong [78] the mechanism of antioxidants activity in emulsions is very complex. Nonpolar or amphiphilic antioxidant protectors with low HLB could be mainly concentrated at the oil-water interface. In effect those substances could form a protective membrane around the lipid droplets, while polar antioxidants could be dissolved in the aqueous phase. On the other hand, according to Hano et al. [8] some antioxidant compounds do not necessarily follow the polar paradox observation. It is an effect of antioxidant compounds’ migration between the nonpolar and polar phases of the emulsions. Probably during the emulsification and spray-drying the part of antioxidant compounds, such as SDG and other polyphenolics, could migrate to the oil fraction. The powder with 20% of oil exhibited lower water activity, lower content of surface free oil, and hygroscopicity. This observation is in line with the findings of Vélez-Erazo et al. who observed that lower moisture content and water activity correspond with a higher oxidative stability of vegetable oil [13]. Thus, this formulation presumably stimulated FOCE components (polyphenolics, proteins, free amino acids) for more effective anti-oxidative protection of FO.

## 4. Conclusions

Taking into account the oxidation susceptibility of FO an approach was applied in this work to use a valuable agro-industrial by-product (FOCE) as a potential antioxidant agent in spray-dried emulsions containing FO. Our study provides an extensive body of evidence that FOCE could be applied to prevent FO in spray-dried powdered emulsions against rancidity throughout four weeks of storage. It could be concluded that the observed effect is caused by the synergistic influence of FOCE compounds (polyphenolics, proteins, amino acids), and changes of their levels resulted from spray-drying. This study could open a promising pathway for producing natural and plant-based spray-dried powders containing valuable flaxseed oil, and further studies on their application in food science should be carried out.

## Figures and Tables

**Table 1:** Water activity (a_w_), hygroscopicity, average particles size of powders, and encapsulation efficiency of flaxseed oil.

Sample	a_w_ (-)	Hygroscopicity (%)	D_4,3_ (µm)	EE (%)
A	0.41 ± 0.01 ^a^	15.48 ± 0.88 ^a^	28.39 ± 0.23 ^b^	22.00 ± 3.24 ^a^
B	0.36 ± 0.00 ^b^	14.75 ± 0.35 ^b^	31.55 ± 0.67 ^a^	39.00 ± 0.02 ^b^

Values are means ± standard deviation of triplicate determinations. Means with different letters in the same column are significantly different at *p* < 0.05.

**Table 2:** Color changes of powders during storage.

L *					
	0	1	2	3	4
A	95.04 ± 0.00 ^Aa^	95.06 ± 0.00 ^Ba^	94.53 ± 0.00 ^Ca^	95.14 ± 0.01 ^Da^	95.22 ± 0.02 ^Ea^
B	94.63 ± 0.00 ^Ab^	94.21 ± 0.00 ^Bb^	94.27 ± 0.00 ^Cb^	94.05 ± 0.00 ^Db^	93.92 ± 0.01 ^Eb^
a *					
A	−1.33 ± 0.00 ^Ab^	−1.42 ± 0.00 ^Bb^	−1.26 ± 0.03 ^Cb^	−1.25 ± 0.02 ^Db^	−1.23 ± 0.00 ^Eb^
B	−1.13 ± 0.01 ^Aa^	−1.09 ± 0.01 ^Ba^	−1.08 ± 0.02 ^Ca^	−1.02 ± 0.00 ^Da^	−1.15 ± 0.00 ^Ea^
b *					
A	9.35 ± 0.01 ^Ab^	10.00 ± 0.01 ^Bb^	8.84 ± 0.03 ^Bb^	8.81 ± 0.01 ^Bb^	8.31 ± 0.01 ^Cb^
B	10.37 ± 0.01 ^Aa^	10.50 ± 0.01 ^Ba^	10.37 ± 0.01 ^Ca^	10.20 ± 0.05 ^Da^	9.67 ± 0.01 ^Ea^
YI					
A	13.06 ± 0.01 ^Ab^	13.02 ± 0.01 ^Bb^	13.36 ± 0.01 ^Cb^	13.23 ± 0.02 ^Db^	13.46 ± 0.08 ^Eb^
B	15.66 ± 0.01 ^Aa^	15.92 ± 0.01 ^Ba^	15.71 ± 0.01 ^Ca^	16.26 ± 0.01 ^Da^	16.72 ± 0.01 ^Ea^
WI					
A	89.33 ± 0.01 ^Aa^	88.76 ± 0.00 ^Ba^	89.53 ± 0.03 ^Ca^	89.86 ± 0.01 ^Da^	89.34 ± 0.05 ^Ea^
B	88.26 ± 0.00 ^Ab^	87.96 ± 0.00 ^Bb^	88.11 ± 0.01 ^Cb^	87.71 ± 0.00 ^Db^	87.96 ± 0.00 ^Eb^
ΔE					
A	Used as a standard	0.65 ± 0.01 ^Aa^	0.73 ± 0.01 ^Ba^	0.67 ± 0.00 ^Ca^	1.06 ± 0.05 ^Da^
B	Used as a standard	0.44 ± 0.01 ^Ab^	0.36 ± 0.01 ^Bb^	0.55 ± 0.01 ^Cb^	0.70 ± 0.01 ^Db^

L *—lightness; a *—redness/greenness; b *—yellowness/blueness; YI—yellowness index; WI—whiteness index; ΔE—total color difference. Values are means ± standard deviation of triplicate determinations. Means with different lowercase letters in the same column are significantly different at *p* < 0.05. Means with different uppercase letters in the same raw are significantly different at *p* < 0.05.

**Table 3:** The antioxidant activity changes of dissolved powders during storage.

ABTS (%)					
Sample	0	1	2	3	4
A	74.15 ± 0.08 ^Aa^	61.47 ± 0.14 ^Bb^	58.22 ± 0.16 ^Cb^	54.50 ± 0.01 ^Da^	53.35 ± 0.08 ^Ea^
B	74.01 ± 0.08 ^Ab^	66.67 ± 0.08 ^Ba^	61.05 ± 0.00 ^Ca^	58.50 ± 0.08 ^Da^	54.21 ± 0.16 ^Eb^
DPPH (%)					
A	65.27 ± 0.05 ^Aa^	58.57 ± 0.00 ^Ba^	54.66 ± 0.03 ^Ca^	52.24 ± 0.06 ^Db^	51.21 ± 0.05 ^Eb^
B	63.03 ± 0.02 ^Ab^	55.24 ± 0.05 ^Bb^	53.39 ± 0.07 ^Cb^	52.67 ± 0.07 ^Da^	51.71 ± 0.02 ^Ea^
TPC (mg GAE/mL)					
A	19.08 ± 0.01 ^Ab^	18.84 ± 0.02 ^Bb^	17.80 ± 0.02 ^Cb^	16.01 ± 0.02 ^Db^	15.06 ± 0.00 ^Ea^
B	20.05 ± 0.01 ^Aa^	19.69 ± 0.05 ^Ba^	18.15 ± 0.02 ^Ca^	17.59 ± 0.00 ^Da^	16.98 ± 0.02 ^Eb^
TFAA (mg Gly/mL)					
Sample	0	1	2	3	4
A	5.74 ± 0.06 ^Ab^	10.68 ± 0.04 ^Bb^	11.19 ± 0.02 ^Cb^	12.23 ± 0.07 ^Db^	12.32 ± 0.04 ^Eb^
B	9.03 ± 0.13 ^Aa^	12.36 ± 0.04 ^Ba^	12.93 ± 0.09 ^Ca^	14.97 ± 0.06 ^Da^	15.50 ± 0.12 ^Ea^

Values are means ± standard deviation of triplicate determinations. Means with different lowercase letters in the same column are significantly different at *p* < 0.05. Means with different uppercase letters in the same raw are significantly different at *p* < 0.05.

## Data Availability

The data presented in this study are available on request from the corresponding authors.

## References

[1] Michotte D., Rogez H., Chirinos R., Mignolet E., Campos D., Larondelle Y. (2011). Linseed oil stabilisation with pure natural phenolic compounds. Food Chem..

[2] Zhang Z.-S., Wang L.-J., Li D., Li S.-J., Özkan N. (2011). Characteristics of Flaxseed Oil from Two Different Flax Plants. Int. J. Food Prop..

[3] Domian E., Brynda-Kopytowska A., Marzec A. (2017). Functional Properties and Oxidative Stability of Flaxseed Oil Microencapsulated by Spray Drying Using Legume Proteins in Combination with Soluble Fiber or Trehalose. Food Bioprocess Technol..

[4] Carneiro H.C.F., Tonon R.V., Grosso C.R.F., Hubinger M.D. (2013). Encapsulation efficiency and oxidative stability of flaxseed oil microencapsulated by spray drying using different combinations of wall materials. J. Food Eng..

[5] Goyal A., Sharma V., Sihag M.K., Arora S., Singh A.K., Sabikhi L. (2016). Effect of microencapsulation and spray drying on oxidative stability of flaxseed oil and its release behavior under simulated gastrointestinal conditions. Dry. Technol..

[6] Gumus C.E., Decker E.A., Mcclements D.J. (2017). Formation and Stability of ω -3 Oil Emulsion-Based Delivery Systems Using Plant Proteins as Emulsifiers: Lentil, Pea, and Faba Bean Proteins. Food Biophys..

[7] Tzang B.S., Yang S.F., Fu S.G., Yang H.C., Sun H.L., Chen Y.C. (2009). Effects of dietary flaxseed oil on cholesterol metabolism of hamsters. Food Chem..

[8] Hano C., Corbin C., Drouet S., Quéro A., Rombaut N., Savoire R., Molinié R., Thomasset B., Mesnard F., Lainé E. (2017). The lignan (+)-secoisolariciresinol extracted from flax hulls is an effective protectant of linseed oil and its emulsion against oxidative damage. Eur. J. Lipid Sci. Technol..

[9] Kchaou H., Jridi M., Nasri M., Debeaufort F. (2020). Design of Gelatin Pouches for the Preservation of Flaxseed Oil during Storage. Coatings.

[10] Herchi W., Bouali I., Bahashwan S., Rochut S., Boukhchina S., Kallel H., Pepe C. (2012). Changes in phospholipid composition, protein content and chemical properties of flaxseed oil during development. Plant Physiol. Biochem..

[11] Ghani M.A., Barril C., Bedgood D.R., Prenzler P.D. (2019). Development of a Method Suitable for High-Throughput Screening to Measure Antioxidant Activity in a Linoleic Acid Emulsion. Antioxidants.

[12] Waszkowiak K., Mikołajczak B. (2020). The Effect of Roasting on the Protein Profile and Antiradical Capacity of Flaxseed Meal. Foods.

[13] Vélez-Erazo E.M., Consoli L., Hubinger M.D. (2020). Spray drying of mono- and double-layer emulsions of PUFA-rich vegetable oil homogenized by ultrasound. Dry. Technol..

[14] Piñón-Balderrama C.I., Leyva-Porras C., Terán-Figueroa Y., Espinosa-Solís V., Álvarez-Salas C., Saavedra-Leos M.Z. (2020). Encapsulation of active ingredients in food industry by spray-drying and nano spray-drying technologies. Processes.

[15] Ancuţa P., Sonia A. (2020). Oil press-cakes and meals valorization through circular economy approaches: A review. Appl. Sci..

[16] Ramachandran S., Singh S.K., Larroche C., Soccol C.R., Pandey A. (2007). Oil cakes and their biotechnological applications—A review. Bioresour. Technol..

[17] Fruehwirth S., Steinschaden R., Woschitz L., Richter P., Schreiner M., Hoffmann B., Hoffmann W., Pignitter M. (2020). Oil-assisted extraction of polyphenols from press cake to enhance oxidative stability of flaxseed oil. LWT.

[18] Drozłowska E., Łopusiewicz Ł., Mężyńska M., Bartkowiak A. (2020). The effect of native and denaturated flaxseed meal extract on physiochemical properties of low fat mayonnaises. J. Food Meas. Charact..

[19] Drozłowska E., Bartkowiak A., Łopusiewicz Ł. (2020). Characterization of Flaxseed Oil Bimodal Emulsions Prepared with Flaxseed Oil Cake Extract Applied as a Natural Emulsifying Agent. Polymers.

[20] Drozłowska E., Łopusiewicz Ł., Mężyńska M., Bartkowiak A. (2020). Valorization of Flaxseed Oil Cake Residual from Cold-Press Oil Production as a Material for Preparation of Spray-Dried Functional Powders for Food Applications as Emulsion Stabilizers. Biomolecules.

[21] Jarzębski M., Smułek W., Siejak P., Rezler R., Pawlicz J., Trzeciak T., Jarzębska M., Majchrzak O., Kaczorek E., Kazemian P. (2021). *Aesculus hippocastanum* L. as a Stabilizer in Hemp Seed Oil Nanoemulsions for Potential Biomedical and Food Applications. Int. J. Mol. Sci..

[22] Cai Y.Z., Corke H. (2000). Production and properties of spray-dried Amaranthus betacyanin pigments. J. Food Sci..

[23] Calvo P., Castaño Á.L., Hernández M.T., González-Gómez D. (2011). Effects of microcapsule constitution on the quality of microencapsulated walnut oil. Eur. J. Lipid Sci. Technol..

[24] Chegini G., HamidiSepehr A., Dizaji M.F., Mirnezami S.V. (2014). Study of physical and chemical properties of spray drying whey powder. Int. J. Recycl. Org. Waste Agric..

[25] Huang C., Wang S., Yang H. (2020). Evaluation of Thermal Effects on the Bioactivity of Curcumin Microencapsulated with Porous Starch-Based Wall Material Using Spray Drying. Processes.

[26] Pan Y., Wu Z., Xie Q.T., Li X.M., Meng R., Zhang B., Jin Z.Y. (2020). Insight into the stabilization mechanism of emulsions stabilized by Maillard conjugates: Protein hydrolysates-dextrin with different degree of polymerization. Food Hydrocoll..

[27] Barac M., Cabrilo S., Pesic M., Stanojevic S., Zilic S., Macej O., Ristic N. (2010). Profile and Functional Properties of Seed Proteins from Six Pea (*Pisum sativum*) Genotypes. Int. J. Mol. Sci..

[28] Tong T., Liu Y.-J., Kang J., Zhang C.-M., Kang S.-G. (2019). Antioxidant Activity and Main Chemical Components of a Novel Fermented Tea. Molecules.

[29] Bligh E.G., Dyer W.J. (1959). A rapid method of total lipid extraction and purification. Can. J. Biochem. Physiol..

[30] Łopusiewicz Ł., Drozłowska E., Tarnowiecka-Kuca A., Bartkowiak A., Mazurkiewicz-Zapałowicz K., Salachna P. (2020). Biotransformation of Flaxseed Oil Cake into Bioactive Camembert-Analogue Using Lactic Acid Bacteria, *Penicillium camemberti* and *Geotrichum candidum*. Microorganisms.

[31] Fioramonti S.A., Stepanic E.M., Tibaldo A.M., Pavón Y.L., Santiago L.G. (2019). Spray dried flaxseed oil powdered microcapsules obtained using milk whey proteins-alginate double layer emulsions. Food Res. Int..

[32] Aberkane L., Roudaut G., Saurel R. (2014). Encapsulation and Oxidative Stability of PUFA-Rich Oil Microencapsulated by Spray Drying Using Pea Protein and Pectin. Food Bioprocess Technol..

[33] Klinkesorn U., Sophanodora P., Chinachoti P., McClements D.J. (2004). Stability and rheology of corn oil-in-water emulsions containing maltodextrin. Food Res. Int..

[34] Fernandes R.V.D.B., Borges S.V., Botrel D.A. (2014). Gum arabic/starch/maltodextrin/inulin as wall materials on the microencapsulation of rosemary essential oil. Carbohydr. Polym..

[35] Lee J.K.M., Taip F.S., Abdulla H.Z. (2018). Effectiveness of additives in spray drying performance: A review. Food Res..

[36] Samsu Z.A., Mohamad Zahir A.Z. (2020). Production of oil palm milk powder by spray drying technique. Mater. Today Proc..

[37] Chang Y.I., Scire J., Jacobs B. (1988). Effect of Particle Size and Microstructure Properties on Encapsulated Orange Oil. Flavor Encapsulation.

[38] Kumar L.R.G., Chatterjee N.S., Tejpal C.S., Vishnu K.V., Anas K.K., Asha K.K., Anandan R., Mathew S. (2017). Evaluation of chitosan as a wall material for microencapsulation of squalene by spray drying: Characterization and oxidative stability studies. Int. J. Biol. Macromol..

[39] Karaca A.C., Low N., Nickerson M. (2013). Encapsulation of Flaxseed Oil Using a Benchtop Spray Dryer for Legume Protein−Maltodextrin Microcapsule Preparation. J. Agric. Food Chem..

[40] Barbosa-Cánovas G.V., Ortega-Rivas E., Juliano P., Yan H. (2005). Food Powders: Physical Properties, Processing, and Functionality.

[41] Tonon R.V., Pedro R.B., Grosso C.R.F., Hubinger M.D. (2012). Microencapsulation of Flaxseed Oil by Spray Drying: Effect of Oil Load and Type of Wall Material. Dry. Technol..

[42] Badke L.B., da Silva B.C., de Carvalho-Jorge A.R., Taher D.M., Riegel-Vidotti I.C., Marino C.E.B. (2019). Synthesis and characterization of microalgae fatty acids or Aloe vera oil microcapsules. Polimeros.

[43] Muzaffar K., Kumar P. (2016). Moisture sorption isotherms and storage study of spray dried tamarind pulp powder. Powder Technol..

[44] Stoll L., Silva A.M.D., Iahnke A.O.E.S., Costa T.M.H., Flôres S.H., Rios A.D.O. (2017). Active biodegradable film with encapsulated anthocyanins: Effect on the quality attributes of extra-virgin olive oil during storage. J. Food Process. Preserv..

[45] Kim J.Y., Yi B.R., Lee C., Gim S.Y., Kim M.J., Lee J.H. (2016). Effects of pH on the rates of lipid oxidation in oil–water system. Appl. Biol. Chem..

[46] Du J., Ge Z.Z., Xu Z., Zou B., Zhang Y., Li C.M. (2014). Comparison of the Efficiency of Five Different Drying Carriers on the Spray Drying of Persimmon Pulp Powders. Dry. Technol..

[47] Tambade P.B., Sharma M., Singh A.K., Surendranath B. (2020). Flaxseed Oil Microcapsules Prepared Using Soy Protein Isolate and Modified Starch: Process Optimization, Characterization and In Vitro Release Behaviour. Agric. Res..

[48] Grehk T.M., Berger R., Bexell U. (2008). Investigation of the drying process of linseed oil using FTIR and ToF-SIMS. J. Phys. Conf. Ser..

[49] Palanisamy K.L., Devabharathi V., Sundaram N.M. (2013). The utility of magnetic iron oxide nanoparticles stabilized by carrier oils in removal of heavy metals from waste water. IJRANS.

[50] Souza C.R.F., Georgetti S.R., Salvador M.J., José M., Fonseca V., Oliveira W.P. (2009). Antioxidant activity and physical-chemical properties of spray and spouted bed dried extracts of Bauhinia forficata. Braz. J. Pharm. Sci..

[51] Malcolmson L.J., Przybylski R., Daun J.K. (2000). Storage stability of milled flaxseed. J. Am. Oil Chem. Soc..

[52] Pag A.I., Radu D.G., Draganescu D., Popa M.I., Sîrghie C. (2014). Flaxseed cake—A sustainable source of antioxidant and antibacterial extracts. Cellul. Chem. Technol..

[53] Gerstenmeyer E., Reimer S., Berghofer E., Schwartz H., Sontag G. (2013). Effect of thermal heating on some lignans in flax seeds, sesame seeds and rye. Food Chem..

[54] Imran M., Ahmad N., Anjum F.M., Khan M.K., Mushtaq Z., Nadeem M., Hussain S. (2015). Potential protective properties of flax lignan secoisolariciresinol diglucoside. Nutr. J..

[55] Ramsay A., Fliniaux O., Quéro A., Molinié R., Demailly H., Hano C., Paetz C., Roscher A., Grand E., Kovensky J. (2017). Kinetics of the incorporation of the main phenolic compounds into the lignan macromolecule during flaxseed development. Food Chem..

[56] Corbin C., Fidel T., Leclerc E.A., Barakzoy E., Sagot N., Falguiéres A., Renouard S., Blondeau J.P., Ferroud C., Doussot J. (2015). Development and validation of an efficient ultrasound assisted extraction of phenolic compounds from flax (*Linum usitatissimum* L.) seeds. Ultrason. Sonochem..

[57] Renouard S., Hano C., Corbin C., Fliniaux O., Lopez T., Montguillon J., Barakzoy E., Mesnard F., Lamblin F., Lainé E. (2010). Cellulase-assisted release of secoisolariciresinol from extracts of flax (*Linum usitatissimum*) hulls and whole seeds. Food Chem..

[58] Socrier L., Quéro A., Verdu M., Song Y., Molinié R., Mathiron D., Pilard S., Mesnard F., Morandat S. (2019). Flax phenolic compounds as inhibitors of lipid oxidation: Elucidation of their mechanisms of action. Food Chem..

[59] Cheng C., Yu X., McClements D.J., Huang Q., Tang H., Yu K., Xiang X., Chen P., Wang X., Deng Q. (2019). Effect of flaxseed polyphenols on physical stability and oxidative stability of flaxseed oil-in-water nanoemulsions. Food Chem..

[60] Shahidi F., Zhong Y. (2015). Measurement of antioxidant activity. J. Funct. Foods.

[61] Durante M., Milano F., De Caroli M., Giotta L., Piro G., Mita G., Frigione M., Lenucci M.S. (2020). Tomato Oil Encapsulation by α-, β-, and γ-Cyclodextrins: A Comparative Study on the Formation of Supramolecular Structures, Antioxidant Activity, and Carotenoid Stability. Foods.

[62] Mrkìc V., Cocci E., Rosa M.D., Sacchetti G. (2006). Effect of drying conditions on bioactive compounds and antioxidant activity of broccoli (*Brassica oleracea* L.). J. Sci. Food Agric..

[63] Martin-Rubio A.S., Sopelana P., Nakashima F., Shibata T., Uchida K., Guillén M.D. (2019). A dual perspective of the action of lysine on soybean oil oxidation process obtained by combining 1H NMR and LC-MS: Antioxidant effect and generation of Lysine-Aldehyde adducts. Antioxidants.

[64] Hwang H., Winkler-Moser J.K., Doll K.M., Gadgil M., Liu S.X. (2019). Factors Affecting Antioxidant Activity of Amino Acids in Soybean Oil at Frying Temperatures. Eur. J. Lipid Sci. Technol..

[65] Sedaghat Doost A., Nikbakht Nasrabadi M., Wu J., A’yun Q., Van der Meeren P. (2019). Maillard conjugation as an approach to improve whey proteins functionality: A review of conventional and novel preparation techniques. Trends Food Sci. Technol..

[66] Akbarbaglu Z., Mahdi Jafari S., Sarabandi K., Mohammadi M., Khakbaz Heshmati M., Pezeshki A. (2019). Influence of spray drying encapsulation on the retention of antioxidant properties and microstructure of flaxseed protein hydrolysates. Colloids Surf. B Biointerfaces.

[67] You L., Zhao M., Cui C., Zhao H., Yang B. (2009). Effect of degree of hydrolysis on the antioxidant activity of loach (*Misgurnus anguillicaudatus*) protein hydrolysates. Innov. Food Sci. Emerg. Technol..

[68] Xu P., Zheng Y., Zhu X., Li S., Zhou C. (2018). L-lysine and L-arginine inhibit the oxidation of lipids and proteins of emulsion sausage by chelating iron ion and scavenging radical. Asian-Australas. J. Anim. Sci..

[69] Filippenko T.A., Gribova N.Y. (2011). Antioxidant activity of amino acids during oxidation of sunflower oil in an emulsion. Pharm. Chem. J..

[70] Gong K.J., Shi A.M., Liu H.Z., Liu L., Hu H., Adhikari B., Wang Q. (2015). Emulsifying properties and structure changes of spray and freeze-dried peanut protein isolate. J. Food Eng..

[71] Chen C., Chi Y.J., Xu W. (2012). Comparisons on the Functional Properties and Antioxidant Activity of Spray-Dried and Freeze-Dried Egg White Protein Hydrolysate. Food Bioprocess Technol..

[72] Koç M., Güngör Ö., Zungur A., Yalçın B., Selek İ., Ertekin F.K., Ötles S. (2015). Microencapsulation of Extra Virgin Olive Oil by Spray Drying: Effect of Wall Materials Composition, Process Conditions, and Emulsification Method. Food Bioprocess Technol..

[73] Codex Alimentarius Commission, FAO, WHO (2011). Standard for Fish Oils.

[74] Nawar W.W., Fennema O.R. (1996). Lipids in Food Chemistry.

[75] Gallardo G., Guida L., Martinez V., López M.C., Bernhardt D., Blasco R., Pedroza-Islas R., Hermida L.G. (2013). Microencapsulation of linseed oil by spray drying for functional food application. Food Res. Int..

[76] Mao X., Chen W., Huyan Z., Sherazi S.T.H., Yu X. (2020). Impact of linolenic acid on oxidative stability of rapeseed oils. J. Food Sci. Technol..

[77] Esterbauer H., Schaur R.J., Zollner H. (1991). Chemistry and biochemistry of 4-hydroxynonenal, malonaldehyde and related aldehydes. Free Radic. Biol. Med..

[78] Shahidi F., Zhong Y. (2011). Revisiting the polar paradox theory: A critical overview. J. Agric. Food Chem..

[79] Porter W.L., Black E.D., Drolet A.M. (1989). Use of Polyamide Oxidative Fluorescence Test on Lipid Emulsions: Contrast in Relative Effectiveness of Antioxidants in Bulk Versus Dispersed Systems. J. Agric. Food Chem..

[80] Frankel E.N., Huang S.W., Kanner J., German J.B. (1994). Interfacial Phenomena in the Evaluation of Antioxidants: Bulk Oils vs Emulsions. J. Agric. Food Chem..

